# Neonatal neurocritical care considerations for prenatally identified neurological disorders

**DOI:** 10.1038/s41390-025-04691-w

**Published:** 2026-01-07

**Authors:** Dawn Gano, James P. Boardman, Sonika Agarwal, Charu Venkatesan, Tomo Tarui, Vann Chau, Courtney J. Wusthoff, Monica E. Lemmon, Barbara Scelsa, Brigitte Vollmer, Sarah B. Mulkey, Mark S. Scher, Anthony R. Hart, Eric S. Peeples, Firdose Nakwa, Eleanor Molloy, Andrea C. Pardo

**Affiliations:** 1 Department of Pediatrics, BC Children’s Hospital Research Institute, The University of British Columbia, Vancouver, BC Canada; 2 Department of Neurology, University of California, San Francisco, CA USA; 3 Centre for Reproductive Health, Institute for Regeneration and Repair, University of Edinburgh, Edinburgh, UK; 4 Division of Neurology, Perelman School of Medicine at the University of Pennsylvania, Philadelphia, PA USA; 5 Department of Pediatrics, Division of Neurology, Cincinnati Children’s Hospital Medical Center, University of Cincinnati, Cincinnati, OH USA; 6 Division of Pediatric Neurology, Hasbro Children’s, Warren Alpert Medical School of Brown University, Providence, RI USA; 7 Department of Pediatrics (Neurology), The Hospital for Sick Children and University of Toronto, Toronto, ON Canada; 8 Department of Neurology, University of California Davis, Sacramento, CA USA; 9 Departments of Pediatrics and Population Health Sciences, Duke University School of Medicine, Durham, NC USA; 10 Department of Pediatric Neurology, Buzzi Children’s Hospital, Milan, Italy; 11 Clinical Neurosciences, Clinical and Experimental Sciences, Faculty of Medicine, University of Southampton, Southampton, UK; 12 Paediatric and Neonatal Neurology, Southampton Children’s Hospital, University Hospital Southampton NHS Foundation Trust, Southampton, UK; 13 Zickler Family Prenatal Pediatrics Institute, Children’s National Hospital, Washington, DC USA; 14 Departments of Neurology and Rehabilitation Medicine, and Pediatrics, The George Washington University School of Medicine and Health Sciences, Washington, DC USA; 15 Departments of Pediatrics and Neurology, Case Western Reserve University School of Medicine, Cleveland, OH USA; 16 Department of Paediatric Neurology, King’s College Hospital NHS Foundation Trust, London, UK; 17 Department of Pediatrics, University of Nebraska Medical Center, Children’s Nebraska, Child Health Research Institute, Omaha, NE USA; 18 Department of Paediatrics and Child Health, Faculty of Health Sciences, University of the Witwatersrand, Johannesburg, South Africa; 19 Discipline of Paediatrics, School of Medicine, Trinity College Dublin, the University of Dublin, Dublin, Ireland; 20 Trinity Translational Medicine Institute (TTMI), St James Hospital, Dublin, Ireland; 21 Trinity Research in Childhood Centre (TRiCC), Dublin, Ireland; 22 Paediatrics, Coombe Hospital, Dublin, Ireland; 23 Neurodisability, Children’s Health Ireland (CHI), Dublin, Ireland; 24 Neonatology, Children’s Health Ireland (CHI), Dublin, Ireland; 25 Department of Pediatrics (Neurology and Epilepsy), Northwestern University Feinberg School of Medicine, Chicago, IL USA; 26 Department of Neonatology, The Pediatrix Center for Research, Education, Quality and Safety, The Woman’s Hospital of Texas, Sunrise, FL USA; 27 Division of Neonatology, Cleveland Clinic Children’s Hospital, Cleveland, OH USA; 28 Centre for Clinical Brain Sciences, University of Edinburgh, Edinburgh, UK; 29 Department of Pediatrics (Neurology), The Hospital for Sick Children, SickKids Research Institute (Neuroscience and Mental Health) and University of Toronto, Toronto, ON Canada; 30 Department of Pediatrics (Neurology), The Hospital for Sick Children, SickKids Research Institute (Child Health Evaluative Sciences), University of Toronto, Toronto, ON Canada; 31 Division of Neonatology, Mass General Brigham for Children and Harvard Medical School, Boston, MA USA; 32 Department of Pediatrics, Section of Neonatology Cumming School of Medicine, University of Calgary, Calgary, AB Canada; 33 University College London Hospitals, London, England

## Abstract

**Abstract:**

With increased access to advanced prenatal neuroimaging and genetic testing, neurological disorders such as brain malformations, brain injuries, and genetic disorders, are increasingly being diagnosed during pregnancy. In this review, we address neonatal neurocritical care considerations for the population with prenatally identified neurological disorders. We identify antenatal considerations, including planning location of delivery, as well as postnatal considerations, including clinical phenotyping, neuromonitoring, neuroimaging, and genetic testing. The importance of interdisciplinary collaboration between obstetrics, maternal-fetal medicine, neonatology, pediatric neurology, neuroradiology, genetics, palliative care, early intervention and habilitative services is emphasized. We outline high-priority research gaps, and highlight the need for large, multicenter studies that capture diverse geographies, populations, care practices and settings longitudinally.

**Impact:**

- Fetal neurology is a rapidly evolving field owing to the increased prenatal diagnosis of neurological disorders; however, the natural history of many fetal neurological disorders is not well known.
- We identify interdisciplinary neonatal neurocritical care considerations for newborns with prenatally diagnosed neurological disorders, such as neuroimaging, neuromonitoring, and family support.
- We outline high-priority research gaps in fetal neurology relevant to neurocritical care, including the need to prioritize large-scale longitudinal studies on the etiologies, short- and long-term outcomes of fetal neurologic disorders across diverse geographies and populations to improve counseling and care.

## Introduction

Fetal neurology is a rapidly evolving field, and comprises a core component of Neonatal Neurocritical Care, which is gaining recognition as a subspecialty.^1^ Increased access to advanced prenatal genetic testing and fetal brain magnetic resonance imaging (MRI) has enhanced the ability to diagnose neurological disorders in utero.^2,3^ Despite increased prenatal recognition of central nervous system (CNS) malformations and brain injuries, as well as neurogenetic, neurometabolic, and neuromuscular disorders, established prenatal therapies for congenital neurologic conditions are currently limited to in utero repair of myelomeningocele.^4,5^ Prenatal treatment for maternal infection with toxoplasmosis is recommended,^6^ with emerging data that valacyclovir in maternal cytomegalovirus (CMV) infection significantly reduces the rate of congenital CMV (cCMV) diagnosis and symptomatic cCMV at birth.^7,8^ Therapies in the research pipeline foreshadow the opportunity to improve survival and longer-term outcomes.^9^ For instance, fetal embolization of vein of Galen malformation^10,11^ and prenatal treatment of spinal muscular atrophy with risdiplam^12^ have recently been reported. Prenatal treatment of inborn errors of metabolism to correct deficiencies, such as D-serine for 3-phosphoglycerate dehydrogenase deficiency,^13^ are also increasingly being reported. Currently, a Phase 1 study of in utero enzyme replacement therapy for lysosomal storage disorders is underway, with a case report of successful prenatal treatment of infantile-onset Pompe disease.^14^ These cases indicate the potential for markedly improved outcomes with early identification and prenatal intervention.

With diagnostic technology advancing more quickly than the natural history literature, there are limited data on pregnancy, neonatal, and childhood outcomes following prenatal presentation with a neurological disorder, even for more well-characterized conditions such as fetal ventriculomegaly^15^ and agenesis of the corpus callosum.^16^ In the setting of a prenatally identified neurological disorder, a small proportion of newborns will require resuscitation and critical care after delivery. This review addresses neonatal neurocritical care considerations following prenatal presentation with a neurological disorder and highlights the current research gaps pertaining to neonatal neurocritical care in this population.

## Antenatal care considerations

Planning for potential perinatal and postnatal care needs is an important aspect of interdisciplinary prenatal care.^3^ Fetuses with complex brain malformations, neuromuscular disorders, genetic and syndromic conditions, and congenital anomalies such as congenital heart disease may be more likely to need resuscitation at delivery, as well as neonatal critical care and complex care coordination. Prenatal consultation with maternal-fetal medicine, neonatology and pediatric neurology (or a neonatal neurocritical care specialist when available), in addition to other specialty care providers such as neurosurgery, enables the development of individualized care plans.^3,17^ When there is uncertainty about the diagnosis, potential for survival, and/or neurologic outcome, palliative care teams can provide additional support around communication, decision making, the development of holistic management plans, and bereavement.^18^ Shared decisions and recommendations may include delivery location to ensure access to the anticipated level of care that will be required for the maternal-fetal dyad, as well as mode of delivery, plans for interventions at the time of birth, and parental feeding preference.

Considerations for location of delivery should include the level of intensive care available, as well as access to pediatric subspecialties, including neurosurgery, and resources such as electroencephalography (EEG) and MRI.^3,17^ The necessity for neonatal intensive care may be certain in some instances, such as in hydrocephalus secondary to congenital aqueductal stenosis or vein of Galen malformation with hydrops fetalis and cardiac failure, though a spectrum of uncertainty regarding postnatal care needs is more common for prenatally identified neurological disorders. In addition to the likelihood of requirement for critical care, when planning the location of delivery, the feasibility of transport to a higher level of care as needed, and the possibility of maternal separation should also be considered. Delivery in parents’ home community should be considered if critical care is unlikely to be required beyond baseline population-level risks, and postnatal investigation, such as brain MRI, is not clinically necessary shortly after birth. While postnatal MRI may be recommended for many fetal neurologic diagnoses, these are typically recommended within the initial weeks or months and may be performed without sedation or anesthesia.^15,16,19–22^ Parent preference should also be factored into determining the most appropriate location for delivery.

Recommendations regarding mode of delivery are predominantly based on obstetric considerations when fetal neurological conditions are present.^17^ With fetal hydrocephalus, cesarean section delivery may be indicated due to macrocephaly resulting in cephalopelvic disproportion.^15^ With progressive enlargement of the fetal head, the potential for a vaginal delivery diminishes with greater likelihood of a planned cesarean section,^23^ thereby increasing future maternal obstetric risks.^24^ Cesarean section may plausibly have a favorable risk-benefit ratio in other specific patient groups, such as fetal intracranial hemorrhage. While centers may recommend cesarean section delivery for fetal intracranial hemorrhage regardless of etiology, current obstetric guidelines only specifically recommend cesarean section delivery in cases of fetal neonatal autoimmune thrombocytopenia.^25^ Further study is needed to determine whether vaginal delivery is associated with extension or worsening of fetal intracerebral hemorrhage, particularly in relation to the underlying etiology and whether it is monophasic or multiphasic.^19^ In cases of myelomeningocele the decision to plan cesarean section delivery may depend on whether a prenatal repair was performed. While delivery in prenatally repaired myelomeningocele can proceed based on obstetric indications, in unrepaired defects, cesarean section is commonly recommended to avoid trauma to the exposed neural tube defect.^26^ For encephaloceles, cesarean section is typically necessary for larger defects, particularly when located occipitally, and open defects.

Emerging paradigm-changing treatments for neurogenetic conditions will increasingly warrant consideration in antenatal and neonatal care planning. As early diagnosis in utero provides an opportunity for early pre-symptomatic interventions postnatally, complex logistics to obtain and administer treatments in a timely manner postnatally must be addressed. For instance, prenatal diagnosis of spinal muscular atrophy now raises a unique challenge to provide gene replacement therapy as soon as possible after the baby is born, potentially optimizing neurological outcomes.^12,27,28^ As the frontier of precision medicine evolves with the development of disease-specific gene replacement therapies and small-molecule treatments, location of delivery and care coordination across the perinatal age spectrum may have time-sensitive implications for treatment, as well as outcome.

Parental consultation with a perinatal psychologist can help support the difficult and complex emotions that parents may feel in navigating a fetal neurological condition. For prenatal diagnoses with low survival rates or uncertain neurological prognosis, prenatal counseling with perinatal palliative care and neonatology can enhance the provision of holistic, supportive care, and guide expectant parents through complex medical decision-making while aligning with their goals of care.^18,29^ Palliative care team involvement should not be limited to diagnoses felt to be terminal and can be delivered alongside treatments aimed at long-term survival.^18^ Planning management in the prenatal period, such as intensity of resuscitation after delivery, provides an opportunity for care to be aligned with expectant parents’ goals and values, and can avoid inappropriate treatments. Each of these management branch points represent different clinical and parenting decisions. Prenatal communication and counseling for fetal neurological disorders is discussed in detail in a recent review by Field et al.^30^

## Neonatal neurocritical care considerations

Neonates with a prenatal diagnosis of a neurological or genetic disorder may have a range of clinical care needs. Some prenatally diagnosed neurologic conditions like intraventricular hemorrhage (IVH) and congenital infection are associated with increased risk for prematurity.^19,31^ Prematurity and other perinatal risk factors such as hypoxia can further worsen the long-term outcome of conditions diagnosed during the fetal period. Medical complexity due to multisystem organ involvement will also interact with neurological status. There are limited data on perinatal outcomes for a variety of fetal neurological disorders, including preterm birth, growth restriction, need for resuscitation, predictors of needing ventilatory support and mode of feeding. Such data are urgently needed to inform counseling and care.

### Clinical examination and oral feeding

Postnatal phenotyping is an important aspect of fetal neurological follow-up, with the initial clinical examination representing the first opportunity to conduct a physical examination of the patient.^2,3^ When the etiologic diagnosis is unknown prenatally, postnatal examination can inform further work-up and may resolve diagnostic uncertainty in some cases.^32^ Detailed assessment includes thorough examination for dysmorphisms, anomalies that may not have been identified prenatally, neurocutaneous features, signs of a neural tube defect, skull shape and sutures, as well as measurement of growth parameters. A complete neurological examination of alertness and behavior, cranial nerves, movements, posture, muscle tone, strength, deep tendon reflexes, primitive reflexes, and sensation should be performed as feasible based on the newborn’s clinical status. Some clinical examination findings may correlate with developmental or acquired structural abnormalities in a localization-related fashion, such as generalized hypotonia with cerebellar anomalies, dyskinesias with basal ganglia lesions and somnolence with thalamic lesions.

Serial examinations, including assessment of oral feeding ability, can help guide prognosis, regardless of whether an underlying etiologic diagnosis is confirmed. Requirement for tube feeding may be a predictor of motor impairment in prenatally diagnosed brain malformations, neuromuscular and other genetic disorders. In a retrospective cohort of neonates with prenatally identified Dandy-Walker malformation and variants, requirement for gastrostomy tube was a predictor of poor neuromotor development and a non-ambulatory outcome.^33^ Given the small number of cases in this study, it remains uncertain whether feeding difficulty in the neonatal period is an accurate and generalizable marker of severity of motor impairment across congenital brain malformations and genetic disorders. Recent data on fetal intraparenchymal hemorrhage^34^ also point to requirement for gastrostomy tube feeds as a marker of more severe neurodevelopmental impairment. Inadequate oral intake after discharge from the birth hospitalization can also occur as caloric needs increase with growth and the suck reflex is lost, leading to failure to thrive as well as risk for aspiration.

### Interdisciplinary care

Interdisciplinary care needs will depend on the constellation of prenatally diagnosed radiologic findings, underlying diagnosis, extra-uterine adaptation, as well as the postnatal assessment. Care considerations for specific neurological conditions that are commonly diagnosed prenatally and may require neonatal neurocritical care are described in Table 1. Recent review articles on fetal ventriculomegaly,^15^ fetal callosal disorders,^16^ prenatally identified absent septum pellucidum,^20^ fetal malformations of cortical development,^21^ holoprosencephaly,^22^ and fetal intracerebral hemorrhage^19^ provide detailed discussion of prenatal counseling and postnatal management recommendations from the pediatric neurology perspective.

**Table 1:** Neonatal care needs for selected prenatal neurologic diagnoses.

Diagnosis	Clinical Care Considerations	Interdisciplinary Care Needs
Open neural tube defect	- Bowel/bladder care -Antibiotic prophylaxis -Monitor for clinical signs of increased intracranial pressure (ICP) with daily head circumference, examine fontanelle/sutures -Wound/skin care -Assess feeding	-Pediatric Neurosurgery -Pediatric Urology -Consider Pediatric Orthopedics and Physiatry -Physical Therapy and Occupational Therapy
Corpus callosum abnormalities and/or absence of septum pellucidum	-Monitor glucose and electrolytes, rule out hypopituitarism -At risk for neonatal seizures -Assess feeding	-Pediatric Neurology -Pediatric Ophthalmology -Genetics -Pediatric Endocrinology -Physical Therapy and Occupational Therapy
Vein of Galen malformation or other cerebrovascular malformations causing high-output cardiac failure	-Evaluate, monitor and support cardiorespiratory status -Monitor for clinical signs of increased ICP -Monitor for consumptive coagulopathy and thrombocytopenia -At risk for neonatal seizures -Assess feeding	-Interventional Neuroradiology and/or Pediatric Neurosurgery -Pediatric Neurology -Pediatric Cardiology -Physical Therapy and Occupational Therapy -Consider Palliative Care
Tuberous sclerosis complex (TSC)	-At risk for neonatal seizures -Evaluate and monitor cardiorespiratory status -Assess for systemic features of TSC including ophthalmologic and renal -Assess feeding	-Pediatric Neurology -Genetics -Pediatric Cardiology, and Pediatric Nephrology based on organ involvement -Consider Pediatric Ophthalmology, and Dermatology as part of inpatient care or referral for outpatient evaluation -Physical Therapy and Occupational Therapy
Severe congenital ventriculomegaly or hydrocephalus	-Monitor for clinical signs of increased ICP -At risk for neonatal seizures, particularly if non-isolated -Assess feeding	-Pediatric Neurology -Pediatric Neurosurgery -Genetics -Baseline ophthalmologic exam -Physical Therapy and Occupational Therapy -Consider Palliative Care
Congenital neuromuscular disorder (e.g., spinal muscular atrophy, congenital myotonic dystrophy, congenital myopathy)	-Evaluate and monitor cardiorespiratory status -Disease-specific therapy where available, e.g., spinal muscular atrophy -Assess feeding	-Pediatric Neurology -Genetics -Pediatric Cardiology if cardiac involvement -Physical Therapy and Occupational Therapy -Consider Palliative Care
Congenital infection with brain injury and/or disruptive malformation	-Evaluate for multisystem organ involvement -At risk for neonatal seizures -Infection-specific treatment where available, e.g., cytomegalovirus, herpes simplex virus 1 and 2 -Assess feeding	-Pediatric Infectious Disease -Pediatric Neurology -Pediatric Ophthalmology -Audiology/ENT -Physical Therapy and Occupational Therapy

### Neuromonitoring

Children with congenital brain malformations are at risk for the development of epilepsy, defined as a tendency toward unprovoked seizures; however, which neonates are at the highest risk for the earliest onset of seizures is not well known.^35,36^ Malformations of cortical development account for 25–40% of drug-resistant epilepsy in childhood, with the onset of seizures as early as one day of age,^35–37^ and in rare cases in utero.^38^ Existing studies on seizure outcomes in fetuses with congenital brain malformations have been limited in scope and size, and by retrospective design. The large prospective cohort of fetal brain MRI findings in the United Kingdom, the MERIDIAN study, did not report seizures or epilepsy outcomes.^39,40^ A single-center retrospective study showed that neonatal-onset epilepsy was diagnosed in 18/132 (14%) newborns with brain malformations identified prenatally or postnatally.^35^ The highest prevalence of epilepsy was in newborns with malformations of cortical development and importantly, seizures were exclusively electrographic in 28%, emphasizing the role of continuous EEG monitoring.^35^ In contrast, a contemporary retrospective cohort study of neonatal outcomes in common fetal brain MRI abnormalities found neonatal seizures in 2/94 (2%), though only a minority of cases had cortical malformations (8%) or prosencephalic anomalies (6%).^41^ Both studies highlighted a high mortality rate among newborns with congenital brain malformations that develop seizures shortly after birth, indicating a need to identify this high-risk group to inform prenatal counseling and postnatal care.

Fetal onset of seizures is poorly understood. A recent review identified 29 reported cases of fetal seizures in the literature diagnosed clinically and/or by ultrasound.^38^ The mortality rate was 15/24 (63%) in liveborn cases and neurodevelopmental delay or impairment occurred in 67% of the 12 cases that survived beyond the neonatal period. Pyridoxine-dependent epilepsy occurred in 4/29 (14%) reported cases, indicating the importance of biochemical and genetic testing for this treatable condition with early-onset seizures, including in utero.^38^ As the onset of seizures can occur any time beyond the neonatal period in infants and children with congenital brain malformations, brain injury, and genetic disorders, surveillance for seizures is a component of long-term follow-up in this population.

The American Clinical Neurophysiology Society (ACNS) recommends continuous video-EEG in newborns at high-risk for seizures, including those with evidence of brain malformation on neuroimaging if there are clinical concerns for seizures.^42^ Amplitude-integrated EEG (aEEG) is a validated tool for seizure detection in the neonatal period, including in newborns with brain malformation, though the potential yield of aEEG in neonates with brain malformations is not well known compared to those with structurally normal brain development. In a single-center study of neonatal aEEG in 105 newborns,^43^ 3/10 participants with brain malformations had seizures; however, the small number with congenital malformations limited evaluation of the predictive value of aEEG for EEG findings, as well as one-year neurodevelopmental outcome.

The International League Against Epilepsy Task Force on Neonatal Seizures recommends phenobarbital as the first-line anti-seizure medication for neonatal seizures, and notes that special consideration should be given to anti-seizure medication selection based on the prenatal neurologic and genetic history.^44^ Sodium channel blockers are preferred as first-line treatment for neonates with a suspected channelopathy, such as *SCN2A* or *SCN8A*, typically suggested by the family history. Carbamazepine is also effective in *KCNQ2*.^45^ Other channelopathies involving potassium or calcium channels may benefit from different treatment approaches. In cases of complex brain malformations with neonatal onset of seizures,^32^ balancing the seizure burden with medication side effects may require a different goal than seizure freedom. Caution regarding the use of valproate for neonatal seizures is also recommended, and valproate should be avoided in the setting of suspected metabolic disorders and hepatic derangement.

Treatment of seizures in newborns with congenital brain malformations or neurological disorders should include assessment for provoking factors, such as infection, electrolyte disturbance, and hypoglycemia. In the setting of midline malformation, including absent septum pellucidum, agenesis of the corpus callosum, and holoprosencephaly, hypopituitarism can underlie electrolyte abnormalities and hypoglycemia. Diabetes insipidus can occur in conjunction with hypopituitarism, particularly when the posterior pituitary or hypothalamic function is affected.^46^ If provoking causes are not identified, long-term anti-seizure medication treatment is anticipated when a congenital brain malformation is associated with neonatal seizures.

### Neonatal encephalopathy and congenital neurological disorders

**Fig. 1:**
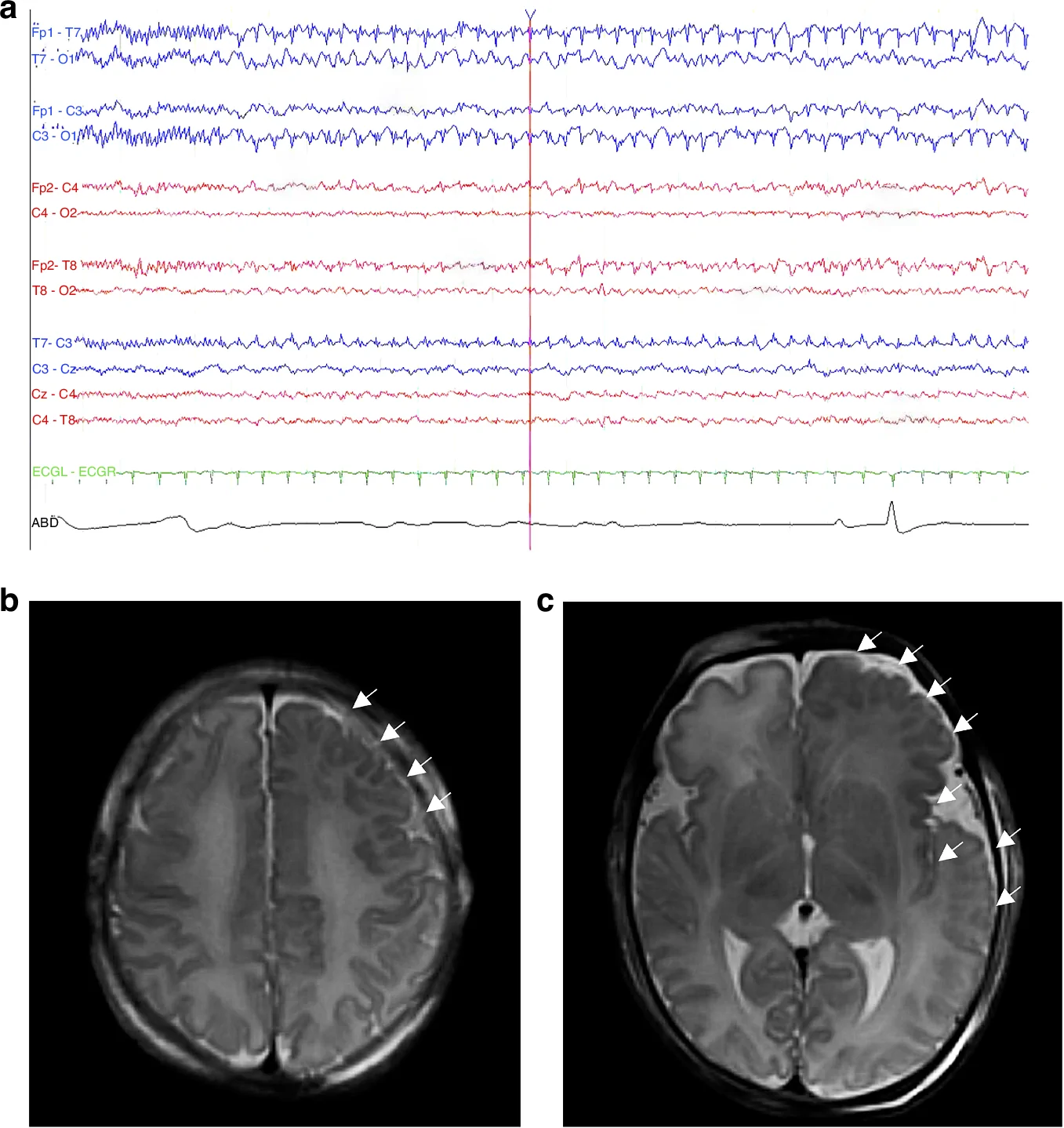
Term infant with hemimegalencephaly, initially presenting with neonatal encephalopathy and treated with therapeutic hypothermia for possible hypoxic-ischemic encephalopathy. **a** Electroencephalography on day 3 showing seizure arising from the left frontal region with spread to the left hemisphere. **b**, **c** Axial T2-weighted MRI after rewarming showing asymmetric enlargement of the left hemisphere with frontotemporal polymicrogyria (white arrows) and hyperintense white matter. Whole exome sequencing was negative; however, genetic testing on resected tissue following hemispherotomy showed a variant in *MTOR*. Routine prenatal anatomy ultrasound was normal in this case, and fetal brain MRI was not obtained.

Neonatal encephalopathy in the population with congenital neurological disorders merits discussion because it can co-exist due to seizures, metabolic disorders or disturbances, or unrelated diagnoses like sepsis.^47^ Hypoxic-ischemic encephalopathy (HIE) has been documented in neonates with congenital neuromuscular disorders and brain malformations in observational cohort studies and clinical trials.^48–52^ For example, an early case series of congenital myotonic dystrophy reported that 13/14 neonates had evidence of perinatal HIE with poor fetal to neonatal transition.^48^ The diagnosis of neonatal encephalopathy may precede the diagnosis of a congenital neurological disorder, which may be identified on brain MRI after rewarming from therapeutic hypothermia (Fig. 1) or based on neurological examination findings that are not explained by the infant’s clinical course, EEG, or MRI.^49–51^ A case series of term neonates with encephalopathy that underwent therapeutic hypothermia for suspected HIE reported 8/137 (6%) cases treated over the study period were subsequently diagnosed with a congenital anomaly or genetic disorder.^49^ In the Phase 2 trial of erythropoietin plus therapeutic hypothermia for perinatal HIE,^50^ 2/50 (4%) participants were diagnosed with another neurological disorder after completing hypothermia. In the randomized Phase 3 HEAL trial of erythropoietin plus therapeutic hypothermia for perinatal HIE,^52^ 24/500 (5%) were found to have a congenital anomaly or genetic disorder, of which there was a wide range of conditions with no apparent linkage to HIE.^51^ There have been no safety concerns in the cases of brain malformations and genetic disorders treated with therapeutic hypothermia for HIE reported in the literature, and it is possible for two conditions to co-occur. HIE in addition to an underlying congenital anomaly or genetic disorder may also further impact neurodevelopmental outcome depending on whether there is resulting acute perinatal injury. The brain malformation or genetic disorder may also increase risk for HIE, so it is important to consider this when evaluating patients with neonatal encephalopathy.

Clinicians may consider a different balance of risks and benefits of therapeutic hypothermia for suspected perinatal HIE in the setting of prenatally diagnosed structural brain malformations and genetic disorders. Whether therapeutic hypothermia is similarly beneficial in neonates with congenital neurological disorders is not known, and may depend on the underlying mechanism for HIE, as well as the specific underlying neurological disorder. For neonates with prenatally recognized neurological disorders that otherwise meet institutional criteria for therapeutic hypothermia, the potential risks and benefits of therapeutic hypothermia should be considered on a case-by-case basis in interdisciplinary discussion specifically between neonatology and pediatric neurology. In the absence of an established contraindication to therapeutic hypothermia, such as a severe congenital anomaly like critical congenital heart disease or congenital diaphragmatic hernia, the potential benefits of therapeutic hypothermia are likely to outweigh the potential risks unless the underlying condition or malformation is associated with an unequivocally severe neurological outcome.

### Neuroimaging

Neuroimaging is an important aspect of postnatal phenotyping in newborns with prenatally identified neurological disorders. The type and timing of neuroimaging will depend on the prenatal findings, as well as clinical status. In cases with prenatally identified ventriculomegaly, particularly when severe ( > 15 mm atrial diameter), non-isolated, and associated with features indicating high-risk for progression (i.e., hemorrhage, aqueductal stenosis), then cranial ultrasound should be obtained soon after delivery.^15^ Cranial ultrasound should also be obtained in cases of prenatally identified intracranial hemorrhage without ventriculomegaly, whether intracerebral (i.e., germinal matrix-intraventricular or intraparenchymal) or extracerebral (i.e., epidural, subdural, subarachnoid or subpial).^19^

**Fig. 2:**
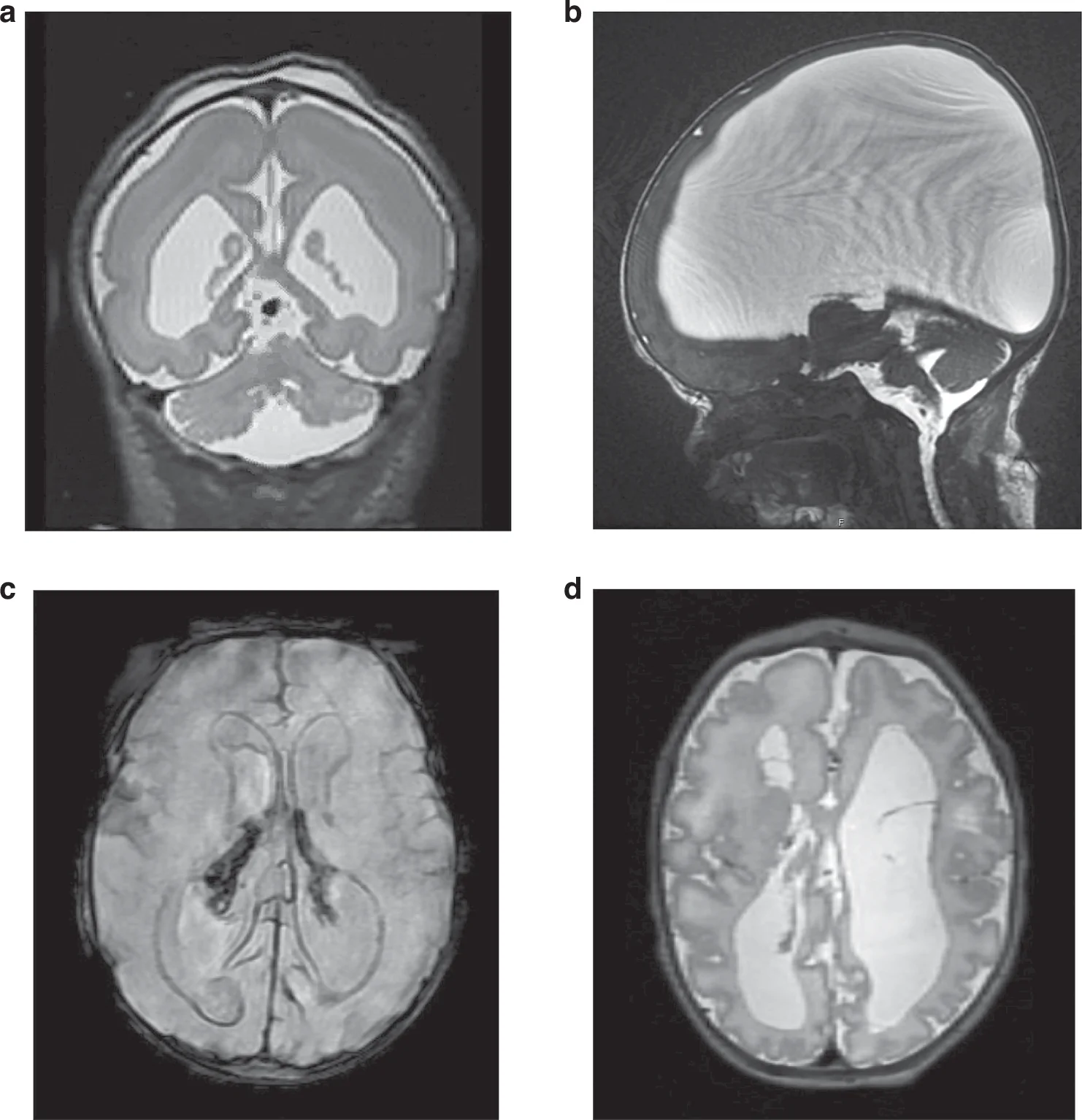
Examples of neonatal MRI findings in selected genetic disorders with initial prenatal presentation. **a** Coronal T2-weighted MRI in newborn with *TUBA1A* variant showing pachygyria-lissencephaly spectrum, ventriculomegaly, and cerebellar hypoplasia. Prenatal presentation in this case with ventriculomegaly on 38-week ultrasound for growth. **b** Sagittal T2-weighted MRI in newborn with *L1CAM* variant showing very severe ventriculomegaly due to aqueductal stenosis. Initial prenatal presentation with mild ventriculomegaly on anatomy ultrasound at 21 gestational weeks and progressive ventriculomegaly on ultrasound throughout pregnancy. **c** Susceptibility-weighted MRI in newborn with *COL4A1* variant and prenatal diagnosis of intraventricular hemorrhage and right posterior periventricular hemorrhagic infarction with ventriculomegaly. Initial diagnosis on fetal brain MRI obtained at 37 gestational weeks for ventriculomegaly on follow up growth ultrasound at 35 gestational weeks. **d** Axial T2-weighted MRI in newborn with *PDHA1*-related pyruvate dehydrogenase complex deficiency showing agenesis of the corpus callosum, ventriculomegaly, cystic cavitation of the white matter, and diffusely abnormal sulcation. Prenatal presentation with similar findings on ultrasound and fetal brain MRI at 32 gestational weeks.

Brain MRI is a superior neuroimaging modality following the prenatal identification of a brain malformation, disruptive brain injury, or both.^53^ Postnatal MRI is important for the detection of late-emerging imaging findings, such as malformations of cortical development, which can occur on a primary basis, or secondary to disruptive consequences of brain injury early in brain development, for example, those due to congenital infection, hypoxia-ischemia, and hemorrhage.^21,34,53,54^ Imaging at term-equivalent age is also necessary to examine the severity of malformations of hindbrain development given that the cerebellum undergoes a rapid period of volumetric growth, surface area expansion, and increased complexity during the third trimester. The full extent of brain malformations can be missed on fetal brain MRI, particularly when performed in the late second trimester and early third trimester; understanding the constellation of malformations informs both the etiologic examination, as well as prognosis.^54^ Some patterns of postnatal MRI findings can point toward specific etiologies (Table 2, Fig. 2). Susceptibility-weighted sequences (e.g., susceptibility-weighted imaging [SWI], or gradient echo imaging, [GRE]) to identify hemosiderin/remote hemorrhage should be obtained, and specifically requested if not routinely included. In selected cases, additional sequences may be helpful, including: (a) magnetic resonance arteriography and venography in cases of suspected vascular malformation or parenchymal hemorrhage,^19^ (b) diffusion tensor imaging in dysgenesis of the corpus callosum to assess the extent of callosal development and to exclude subtler variants of holoprosencephaly,^22^ and (c) magnetic resonance spectroscopy (MRS), which can support a diagnosis of inborn errors of metabolism such as mitochondrial disorders with the presence of a lactate peak, non-ketotic hyperglycinemia (NKH) with evidence of an abnormal glycine peak, and peroxisomal disorders with an abnormal double lipid peak.^55^ Imaging acquisition parameters should be protocolized to optimize visualization of the developing brain, and interpreted by neuroradiologists with expertise in the neonatal brain. There is broad consensus to obtain neonatal brain MRI without sedation or anesthesia unless clinically necessary for another indication (i.e., protecting a critical airway).

**Table 2:** Neonatal neuroimaging patterns in selected genetic and metabolic disorders.

Diagnosis	Associated imaging findings
Zellweger spectrum disorder	-Polymicrogyria -Subependymal and germinolytic cysts -T2-hyperintensity of the supratentorial white matter -Lentioculostriate vasculopathy -Double lipid peak on MRS
Pyruvate dehydrogenase deficiency	-Agenesis or dysgenesis of the corpus callosum -Ventriculomegaly -Cavitary lesions of the white matter -Germinolytic cysts -Lactate peak on MRS
Non-ketotic hyperglycinemia	-Symmetric T2-weighted hyperintensity and reduced diffusion of the myelinated white matter at <3 months of age, including the dorsal brainstem, middle cerebellar peduncles, posterior limbs of the internal capsules, bilateral corona radiata, and perirolandic gyri -Diffuse T2-weighted hyperintensity of the supratentorial white matter between 3-12 months of age -Atrophy of the hippocampus, globus pallidus, cerebral cortex, thalamus, and cerebellum -Structural abnormalities can include agenesis or dysgenesis of the corpus callosum, and gyral abnormalities -Glycine peak on MRS
Tubulinopathy	-Dysmorphic, incompletely separated basal ganglia -Agenesis or dysgenesis of the corpus callosum -Brainstem and cerebellar hypoplasia or dysgenesis -Gyral abnormalities including polymicrogyria and lissencephaly
Tuberous sclerosis complex	-Cortical and subcortical tubers, hyperintense on T1-weighted and hypointense on T2-weighted images in those <3 months -Subependymal nodules, hyperintense on T1-weighted and hypointense on T2-weighted images in those <3 months -Transmantle cortical dysplasia -Subependymal giant cell astrocytoma (rare in neonates)
*COL4A1/2*-related disorders	-Hemorrhagic porencephaly -Hemorrhagic encephalomalacia -Schizencephaly and/or polymicrogyria associated with regions of remote hemorrhage -Supratentorial and infratentorial parenchymal hemorrhage -Intraventricular hemorrhage -Non-hemorrhagic white matter injury

A recent systematic review by Arechvo et al. examined the literature on postnatal brain MRI compared with fetal MRI.^56^ Of the 24 studies with a total of 401 participants identified, all were retrospective or prospective case series judged to be at high risk of selection bias. Postnatal MRI confirmed the findings of fetal MRI in 296 (73.8%), refuted the diagnosis on fetal MRI in 24 (6.2%), and found additional abnormalities in 81 (20.2%).^56^ The role of machine-learning and multi-modal MRI techniques, including structural MRI (sMRI), diffusion MRI (dMRI), functional MRI (fMRI), and perfusion MRI (pMRI), are also under investigation to non-invasively quantify structural and functional maturation at whole brain and regional levels across the gestational spectrum and predict childhood outcomes.^57–61^ The utility of these approaches for children with prenatal diagnoses warrants further investigation.

### Genetic testing

While many newborns with prenatally diagnosed brain malformations and neurological disorders may have undergone genetic and infection testing prenatally, many expectant parents defer invasive testing to the postnatal period. Factors driving decisions on the extent of prenatal versus postnatal work-up for fetal neurologic disorders have not been substantively explored but may be influenced by access to genetic counseling and testing, clinician preference, and patient choice. Understanding these reasons is important since variation might reflect inequitable care. The degree of postnatal testing needed will depend on the prenatal testing performed, clinical phenotype and imaging findings, as well as regional access and availability, and parent preference. Genetic testing of the cord blood planned prenatally can expedite diagnostic evaluation and avoid additional venipuncture.

Increasingly broad sequencing is available prenatally, with a recent survey of fetal neurology practice in the United States reporting availability of prenatal exome sequencing in approximately half of centers.^2^ As access and availability of exome and genome sequencing have evolved prenatally and postnatally, broad sequencing is increasingly replacing targeted gene panels.^62^ Some phenotypes may prompt targeted gene testing because suspicion for a specific disorder is so high, such as *COL4A1/2*-related disorders in congenital hemorrhagic porencephaly,^63^ and X-linked *L1CAM* variants in congenital aqueductal stenosis, hydrocephalus and adducted thumbs,^64^ though both of these - and many other genetic variants resulting in neurological disorders - may have atypical presentations that would still be best served with exome or genome sequencing. Rapid genome sequencing has been shown to have a particularly high yield and clinical relevance to neonatology.^65–67^ For instance, data from Project Baby Bear illustrated 74/184 newborns (40%) in a neonatal intensive care unit received a diagnosis by rapid genome sequencing that explained their admission in a median time of 3 days, and that the diagnosis changed care in 58/74 (78%).^65^ In a prospective study of newborns hospitalized across 6 hospitals in the United States, genome sequencing identified a molecular diagnostic variant in 204/400 (51%) participants, directly affecting clinical care for 19% overall and 37% among those with a diagnostic genome.^66^ Moreover, 76% of clinicians in this study viewed genomic testing as useful or very useful in clinical decision-making, regardless of whether the testing was diagnostic.^66^ Clinicians need to be mindful of the limitations of broad sequencing, including disorders that can be missed, namely trinucleotide repeat disorders (i.e., *DMPK* variants in congenital myotonic dystrophy^68^), disorders of methylation, very large deletions or duplications, and mitochondrial DNA variants. New tools in genetic analysis, such as the growing availability of long-read sequencing, RNA sequencing and multi-omics analysis, may improve detection rates of broad sequencing, though such tools are not widely available in all laboratories yet. Future fetal availability of rapid genome sequencing may be important for families who are confronted by challenging shared clinical decisions.

### Other appropriate postnatal investigations

Some additional tests may be urgent to obtain in the neonatal intensive care setting. In cases of fetal intracranial hemorrhage, a complete blood count with differential and coagulation profile should be obtained,^19^ and testing for fetal-neonatal alloimmune thrombocytopenia should be expedited if not obtained prenatally. Other investigations that should be considered postnatally include evaluation for congenital infection. Given availability for treatment of symptomatic neonates with cCMV disease,^69^ and the 3-week time window after birth to confirm positive cCMV infection, a low threshold for CMV testing in a variety of clinical presentations is recommended, unless there is confirmation of an alternative etiology for prenatal diagnoses such as ventriculomegaly, GMH-IVH, malformation of cortical development, abnormal T2-hyperintensity of the white matter, and hindbrain malformation.^15,19^ Other clinical findings that might prompt concern for CMV include intracranial calcifications, better seen on cranial ultrasound and computed tomography than MRI, as well as brain lesions such as temporal pole abnormalities, subependymal cysts, and intraventricular septations.^70^ Systemic findings may include small for gestational age, peritoneal calcifications, hepatosplenomegaly, thrombocytopenia, transaminitis, rash, and failed newborn hearing screen. If the optimal testing window is missed, testing for cCMV can also be performed using preserved dried blood samples spots from newborn screening if available.^71^ Other infectious etiologies such as toxoplasmosis, syphilis, lymphocytic choriomeningitis virus (LCMV), Zika virus, rubella, herpes simplex virus (HSV-1/2), varicella zoster virus, human immunodeficiency virus (HIV) and parvovirus should also be broadly considered, particularly if CMV testing is negative.^70,72,73^ Concerted efforts should be made to collect and examine the placenta and cord for prenatally diagnosed neurological conditions.^3^ A range of placental histopathological lesions can be seen in congenital infections^74^ that includes microcalcifications, decidual or villous vascular malperfusion lesions, and villous dysmaturity. Viral inclusions can also be identified with special cytological staining techniques.

Although inborn errors of metabolism are collectively rare, they are part of the differential diagnosis for many congenital brain malformations and neonatal encephalopathy. Comprehensive discussion of this topic can be found in reviews by Martinello et al.^75^ and Barsh et al.^76^ Findings that may point strongly toward a metabolic disorder can include persistent anion gap metabolic acidosis, hypoglycemia, hyperammonemia, and elevated lactate, associated with clinical features like hydrops, encephalopathy, neonatal seizures, as well as abnormalities in head circumference. Systemic abnormalities such as cataracts, abnormal hair or skin, persistent liver and clotting abnormalities can also suggest inborn errors of metabolism. The family history should address factors such as multiple miscarriages, neonatal death, learning disability, cerebral palsy, and muscle cramps. Classical imaging patterns can point toward specific disorders, like dysgenesis of the corpus callosum and cystic cavitation of the white matter in pyruvate dehydrogenase complex deficiency,^77^ and under-opercularization of the Sylvian fissures in glutaric aciduria type 1.^78^ Peroxisomal disorders can mimic congenital infection, with similar neuroimaging findings that suggest a destructive etiology associated with cortical malformation.^32,79^ Clinically, Zellweger spectrum disorder is the most common peroxisomal disorder encountered in the neonatal period, and biochemical testing should be prompted by dysmorphic features, a wide fontanelle, and hypotonia associated with suggestive imaging findings.^80^ As specific metabolic disorders cannot be accurately identified based upon the clinical phenotype alone, a battery of laboratory investigations is needed when there is clinical suspicion for a metabolic disorder. Along with exome or genome sequencing, first-line investigations for inborn errors of metabolism should include a gas, anion gap, ammonia, glucose, acylcarnitine panel, free and total carnitine, organic acids, plasma amino acids, and very long chain fatty acids.^75^ When refractory seizures are present, additional laboratory tests should include biotinidase, uric acid, purines and pyrimidines, alpha-amino adipic semialdehyde, pipecolic acid, copper, ceruloplasmin, and cerebrospinal fluid amino acids and neurotransmitters.^75^

The differential diagnosis can also be further clarified by evaluation for systemic manifestations. When undertaking evaluation for congenital infection, dilated ophthalmologic exam, abdominal imaging, skeletal survey, and hearing screening are often performed.^70^ In cases of suspected or confirmed *COL4A1/2*-related disorders, it is routine to perform ophthalmologic exam, electrocardiogram, renal ultrasound and serum creatine kinase.^19^ Disease-specific evaluation for systemic manifestations may be further informed by the specific etiology. Examples include echocardiography in a newborn with a diagnosis of infantile-onset Pompe disease, even if the precordial examination is normal, or renal ultrasound in a newborn with tuberous sclerosis complex.

### Autopsy

Bereaved parents who have experienced neonatal loss report that being offered an autopsy is an opportunity to make parenting choices for their child.^81^ Moreover, parents who have experienced neonatal loss feel it is important for all families to be offered an autopsy, rather than clinicians deciding who would or would not be interested. Parents also report that the option for organ or tissue donation could help to build a legacy to honor their child’s memory, and should be discussed in conjunction with autopsy, ideally by the clinical provider that knows both the infant and family best.^81^

Neuropathological examination of infants who do not survive is important to confirm clinical and imaging diagnoses, as well as identify additional structural, cellular and subcellular features that were not previously known.^82^ Understanding genotype-histopathology correlations can also expand our knowledge on disorders that have been identified since the advent of next-generation sequencing.^83^ Post-mortem MRI is also increasingly available and may be useful as a form of minimally-invasive autopsy to obtain additional information about structural brain anomalies and brain volume in fetuses and infants.^84^

## Research gaps

There are innumerable research gaps within the field of fetal neurology that impact the prenatal and postnatal counseling and care of this unique population. Large, multicenter studies capturing diverse geographies, populations, and care practices are needed to address these questions. Recent publications by a working group of pediatric neurologists have proposed guidelines for postnatal management of specific fetal neurological disorders^15,16,19–22^ and argue for efficient and scalable registry studies to better understand the etiologies, and natural history of prenatally presenting neurological disorders. Here, we summarize key research gaps that are salient to neonatal neurocritical care. There is a critical need for patient and public involvement and engagement in research priority setting in this area.

### Care practices

#### Location, timing, and mode of delivery

Examination of differences in care practices, and differences in outcome by care practices are needed, including: (i) preterm delivery for evolving hydrocephalus, (ii) timing of postnatal cerebrospinal fluid diversion in cases of prenatal post-hemorrhagic ventricular dilatation and hydrocephalus, and (iii) cesarean section for fetal intracerebral hemorrhage regardless of etiology. Identifying reliable prenatal predictors of requirement for resuscitation and neonatal critical care would enable more accurate counseling, as well as more informed decisions about location of delivery.

#### EEG and treatment of seizures

With little data on the risk of neonatal seizures by congenital brain malformation type, it is difficult to predict risk of neonatal seizures prenatally. A better understanding of fetal imaging findings and genetic variants associated with neonatal seizures is needed. Determining how the timing of onset of neonatal seizures, and neonatal seizure burden, may correlate with longer term neurological outcomes could inform counseling and care. Whether there is a role for neonatal EEG in outcome prognostication in the absence of clinical encephalopathy or clinical events suggestive of seizures should be explored. A better understanding of neonatal, infantile and childhood epilepsy outcomes based on malformation type and molecular cause could also inform anti-seizure medication selection and approach to therapy.

#### Postnatal imaging

Studies that determine the rate of new postnatal findings stratified by cause or category, and gestational age at the time of fetal brain MRI, are needed to inform recommendations on the timing of postnatal imaging. Understanding the value of postnatal imaging from the parent perspective is essential, including when postnatal MRI does not identify new findings. Evidence examining how new postnatal findings may contribute to the accuracy of prognostic counseling is also needed. The role of dMRI, MRS, fMRI and computational analyses in this population requires further study.

### Etiologies

#### Deep phenotyping

Large longitudinal studies with standardized investigation for genetic, infectious, metabolic and environmental causes of fetal brain malformations are needed. The convergence of these exposures on the developing brain is also known as the neural exposome,^85^ and cumulative measurement of these exposures will disentangle etiologies and potentially clarify mechanisms.^86^

#### Regional variability

Studies of diverse geographies are needed to determine whether the etiologies of different congenital brain malformations may vary in different regions, including the prevalence and type of different infectious and environmental exposures, as well as regional prevalence of specific genetic variants, which could inform location-specific guidelines for diagnostic work-up.^87^

### Outcomes

#### Obstetric outcomes

There are limited data on obstetric outcomes in pregnancies with a fetal neurological disorder. Capturing the maternal-fetal dyad more fully, including reasons for pregnancy termination and future pregnancy outcomes, would be informative.

#### Perinatal/neonatal and childhood outcomes

Basic data on gestational age at birth and reasons for prematurity are needed. Prenatal predictors of prolonged ventilatory support, tube feeding, and neonatal mortality are needed for clinical counseling and care planning. Length of hospital stay should also be examined. The long-term motor, cognitive, language, learning, vision, hearing, and epilepsy outcomes of prenatally identified neurologic disorders through childhood are not well known.

#### Parent mental health and family outcomes

The impact of prenatal diagnosis of neurological conditions on parent mental health and family well-being is not known. Interventions that address parent stress and social support should be prioritized.

### Treatment

#### Emerging treatments

Feasibility and safety studies of emerging treatment approaches such as in utero enzyme replacement, and ultrasound guided fetal vein of Galen embolization are underway. Global collaboration in participant recruitment will be needed to undertake further study of safety and benefit given the rarity of these conditions.

Optimal prenatal treatment of prenatally identified maternal and/or congenital infections, including CMV and toxoplasmosis, merits further research. The impact of prenatal treatment with mTOR-inhibitors in tuberous sclerosis related cardiac rhabdomyoma requires further study,^88^ including examination of how this may influence postnatal neuroimaging, epilepsy and neurodevelopmental outcomes, as well as optimal dosing and a better understanding of the risk-benefit ratio,

#### Novel therapies

As cell and gene therapy approaches move from the preclinical to the clinical realm, interdisciplinary collaborative research is needed to examine clinical outcomes, particularly of early and pre-symptomatic gene-therapy with the greatest potential benefit. In tandem, the ethical and economic implications of these novel interventions should be examined.^89^

## Conclusions

Newborns with prenatally diagnosed neurological conditions have varying neonatal care needs that necessitate interdisciplinary care in partnership with families. Large-scale studies on the etiologies and obstetric, perinatal, and neonatal outcomes of fetal neurologic disorders across diverse geographies and populations are urgently needed to address key gaps. Understanding the natural history of several fetal neurologic disorders and identifying prenatally available predictors of preterm birth, requirement for neonatal resuscitation, prolonged ventilatory support, tube feeding, as well as neonatal seizures, mortality, and long-term outcomes will improve the accuracy of prenatal counseling. Gene therapy for early and pre-symptomatic newborns with prenatally diagnosed neurogenetic and metabolic disorders should be investigated. Developing evidence-based neonatal neurocritical care practices for newborns with prenatally diagnosed neurological disorders will require multicenter collaboration and a concerted, global effort.
